# Effect of Remifentanil on Mitochondrial Oxygen Consumption of Cultured Human Hepatocytes

**DOI:** 10.1371/journal.pone.0045195

**Published:** 2012-09-13

**Authors:** Siamak Djafarzadeh, Madhusudanarao Vuda, Jukka Takala, Stephan M. Jakob

**Affiliations:** Department of Intensive Care Medicine, Inselspital, Bern University Hospital and University of Bern, Bern, Switzerland; University of South Alabama, United States of America

## Abstract

During sepsis, liver dysfunction is common, and failure of mitochondria to effectively couple oxygen consumption with energy production has been described. In addition to sepsis, pharmacological agents used to treat septic patients may contribute to mitochondrial dysfunction. This study addressed the hypothesis that remifentanil interacts with hepatic mitochondrial oxygen consumption. The human hepatoma cell line HepG2 and their isolated mitochondria were exposed to remifentanil, with or without further exposure to tumor necrosis factor-α (TNF-α). Mitochondrial oxygen consumption was measured by high-resolution respirometry, Caspase-3 protein levels by Western blotting, and cytokine levels by ELISA. Inhibitory κBα (IκBα) phosphorylation, measurement of the cellular ATP content and mitochondrial membrane potential in intact cells were analysed using commercial ELISA kits. Maximal cellular respiration increased after one hour of incubation with remifentanil, and phosphorylation of IκBα occurred, denoting stimulation of nuclear factor κB (NF-κB). The effect on cellular respiration was not present at 2, 4, 8 or 16 hours of incubation. Remifentanil increased the isolated mitochondrial respiratory control ratio of complex-I-dependent respiration without interfering with maximal respiration. Preincubation with the opioid receptor antagonist naloxone prevented a remifentanil-induced increase in cellular respiration. Remifentanil at 10× higher concentrations than therapeutic reduced mitochondrial membrane potential and ATP content without uncoupling oxygen consumption and basal respiration levels. TNF-α exposure reduced respiration of complex-I, -II and -IV, an effect which was prevented by prior remifentanil incubation. Furthermore, prior remifentanil incubation prevented TNF-α-induced IL-6 release of HepG2 cells, and attenuated fragmentation of pro-caspase-3 into cleaved active caspase 3 (an early marker of apoptosis). Our data suggest that remifentanil increases cellular respiration of human hepatocytes and prevents TNF-α-induced mitochondrial dysfunction. The results were not explained by uncoupling of mitochondrial respiration.

## Introduction

Severe sepsis and septic shock are major causes of death in intensive care patients [1], [2]. The causes of organ dysfunction and failure are unclear, but inadequate tissue perfusion, systemic inflammation, and direct metabolic changes at the cellular level are all likely to contribute [3]–[5]. The liver is a central organ in homeostasis, with vital metabolic and immunological functions. During sepsis, liver dysfunction is common, and contributes to the high mortality observed in these patients [6]–[8]. Nevertheless, the precise mechanisms by which the liver is affected are unclear [9]–[10]. Failure of mitochondria to effectively couple oxygen consumption with energy production has been described in sepsis [11]. The pathogenesis of mitochondrial dysfunction is multifactorial, but nitric oxide (NO) [12]–[14], reactive oxygen species (ROS) overproduction [11], anti-oxidant deficiency [11]–[14] and an increase in inner mitochondrial membrane permeability [15]–[16] are likely to contribute. In addition to sepsis, pharmacological agents used to treat septic patients may contribute to mitochondrial dysfunction [17].

The commonly used sedative drug propofol decreases oxygen consumption in brain synaptosomes [18] and impairs mitochondrial respiration in isolated perfused guinea pig hearts [19]. Hanley et al. showed that halothane, isoflurane and sevoflurane inhibit NADH:ubiquinone oxidoreductase (complex I) of cardiac mitochondria [20].

Remifentanil is used to provide analgesia and sedation in critically ill patients [21]. Remifentanil is a synthetic short-acting opioid analgesic drug and is a specific μ-opioid receptor agonist [22]. The potent μ-opioid activity of remifentanil is antagonised by narcotic antagonists, such as naloxone. Unlike other synthetic opioids which are metabolised in the liver, remifentanil has a short half-life and does not accumulate in the body, but is rapidly metabolised by non-specific blood and tissue esterases to carboxylic acid metabolite, which has 1/4600^th^ the potency of the remifentanil [23], [24].

The effect of remifentanil on hepatic mitochondrial bioenergetics has not yet been studied. The primary objective of the present study was to investigate whether remifentanil regulates mitochondrial function in the cultured human hepatocellular carcinoma cell line (HepG2). We used this cell line because it retains most of the liver-specific proteins, metabolic enzymes and functions of primary human hepatocytes [25]. As examples, HepG2 cells and primary human hepatocytes behave similarly when stimulated to express cytochrome P450 [26], genes involved in RNA processing and mitochondrial function [27], and Phase II enzymes [28], with respect to nicotinic acid transport into cells [29], and in terms of acute-phase protein production when stimulated with interleukins [30]. Furthermore, HepG2 cells are available in large quantities, which makes it possible to perform multiple experiments evaluating biochemical functions of liver cells.

Tumour necrosis factor-α (TNF-α) is one of the important mediators of inflammation in sepsis, and may alter mitochondrial function in human hepatocytes [31]. As a secondary objective, we investigated whether remifentanil interferes with TNF-α-induced mitochondrial dysfunction in cultured HepG2 cells.

## Results

### Cellular oxygen consumption after incubation with remifentanil

**Figure 1:**
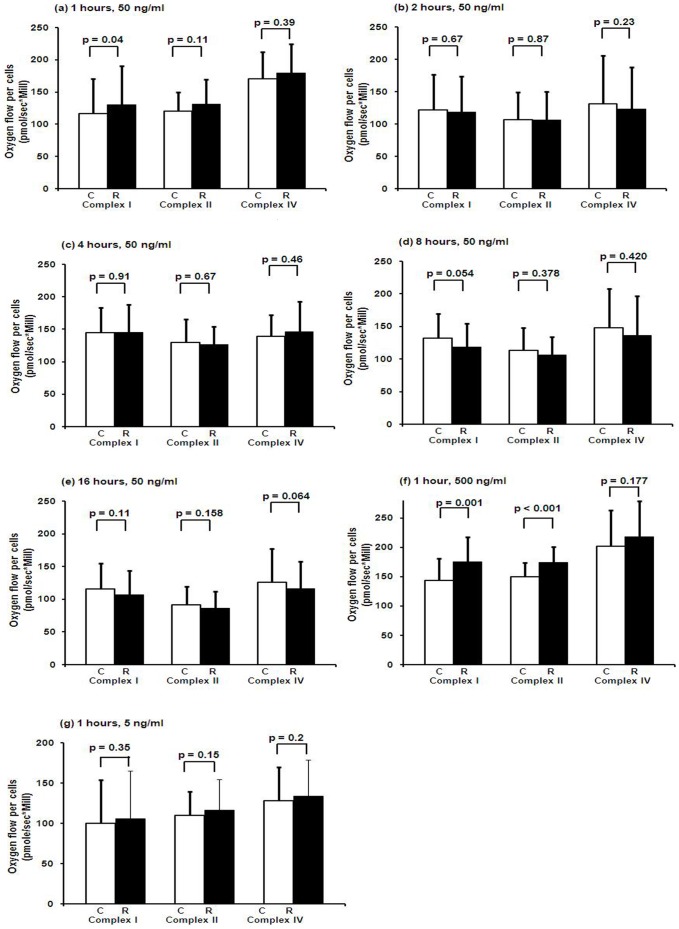
Cellular oxygen consumption after incubation with remifentanil. HepG2 permeabilised cells' oxygen consumption for complex I, II and IV after 1 hour (a) and 2(b), 4(c), 8(d), and 16 hours (e) of incubation with remifentanil at 50 ng/ml (n = 15 each) or after 1 hour of incubation with remifentanil at 500 ng/ml (f) or 5 ng/ml (g) (C: control, R: remifentanil). Controls were incubated with medium alone. Data represent mean±SD. Statistical significance between samples using paired samples *t* test is indicated.

Incubation of HepG2 cells with remifentanil (1 hour, 50 ng/ml; n = 15) induced a significant increase in complex I-dependent respiration (116±54 in controls [incubated in cell culture medium alone] vs. 130±60 pmoles/[s*Million cells] in stimulated cells; p = 0.04), but not complex II- and IV-dependent respiration (Figure 1a). At 1 hour of remifentanil incubation at a concentration of 500 ng/ml (n = 15), a significant increase in respiration was observed for both complex I-dependent respiration (143±38 in controls vs. 175±43 pmoles/[s*Million cells] in stimulated cells; p = 0.001) and complex II-dependent respiration (150±24 in controls vs. 174±27 pmoles/[s*Million cells] in stimulated cells; p<0.001) but not complex IV-dependent cellular respiration (Figure 1f). After 2, 4, 8 and 16 hours of incubation with remifentanil at 50 ng/ml (n = 15 each) and 1 hour at 5 ng/ml, no significant changes in cellular respiration were observed for any of the complexes (Figure 1b–e, 1g). Incubation of the cells with the major component of the vehicle of remifentanil did not affect cellular respiration (data not shown).

### Cellular oxygen consumption after naloxone antagonism

**Figure 2:**
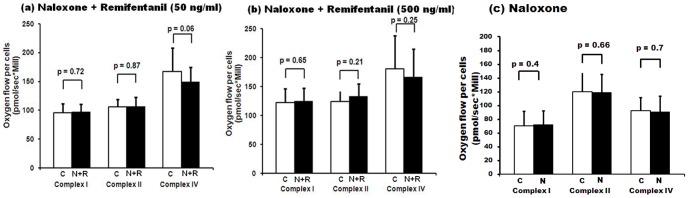
Cellular oxygen consumption after naloxone antagonism. HepG2 permeabilised cells' oxygen consumption for complex I, II and IV. Cells were incubated for 1 hour with medium alone (controls, white bars) or preincubated with naloxone (1000 ng/ml) for 1 hour followed by incubation with remifentanil at 50 ng/ml (Figure 2a) or 500 ng/ml (Figure 2b) for an additional hour (black bars) (n = 15). Figure 2c: Cells were incubated for 2 hour with medium alone (white bars) or incubated with naloxone (1000 ng/ml) for 2 hour (black bars) (n = 10). (C: control, R: remifentanil, N: naloxone). Data represent mean±SD. Statistical significance between samples using paired samples *t* test is indicated.

In order to see if the alterations in mitochondrial respiration could be prevented by the presence of an opioid receptor antagonist, we determined the effect of the antagonist naloxone (Figure 2). For these experiments, the cells were pre-incubated for a 1-hour period with naloxone at a concentration of 1000 ng/ml before the addition of remifentanil (50 or 500 ng/ml; n = 15, Figure 2a, 2b). In another series, the effect of naloxone alone (1000 ng/ml) for 2 hours was compared to medium alone (n = 10; Figure 2C). The controls were incubated with medium alone. Afterwards, cellular respiration was measured. The data indicate that the effect of remifentanil is prevented with naloxone: cells pre-treated with naloxone before the addition of remifentanil exhibited no significant changes in complex activities in comparison with controls. Naloxone alone did not affect mitochondrial respiration (Figure 2c).

### Remifentanil (50 or 500 ng/ml) induces phosphorylation of IκBα

**Figure 3:**
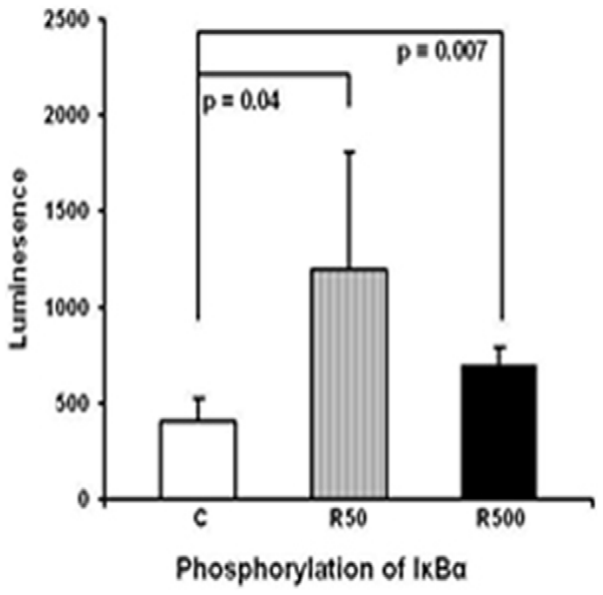
Remifentanil (50 or 500 ng/ml) induces phosphorylation of IκBα. IκBα phosphorylation state is measured in HepG2 cell extract 30 min after stimulation with remifentanil (50 or 500 ng/ml) by a transcription factor Elisa kit (n = 4) (C: control [medium alone], R50: remifentanil 50 ng/ml, R500: remifentanil 500 ng/ml). Data represent mean±SD. Statistical significance between samples using unpaired *t* test is indicated.

To assess the effect of remifentanil (50 or 500 ng/ml) on intracellular signaling, we investigated the phosphorylation state of IκBα. Phosphorylation of IκBα leads to its degradation and results in the release and activation of transcription factor NF-κB. Treatment with remifentanil for 30 min (50 or 500 ng/ml) led to the increased chemiluminescence signal arising from phosphorylation of IκBα (remifentanil 50 ng/ml: 2.9 fold, p = 0.04 and 500 ng/ml: 1.8 fold, p = 0.007) (Figure 3).

### HepG2 isolated mitochondrial oxygen consumption after incubation with remifentanil

**Figure 4:**
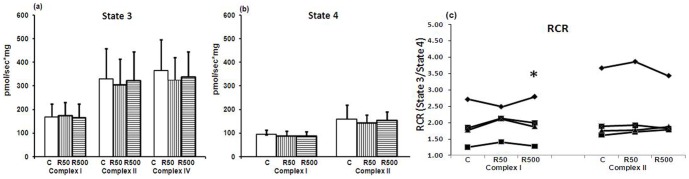
HepG2 isolated mitochondrial oxygen consumption after incubation with remifentanil. HepG2 cells' isolated mitochondrial oxygen consumption for complex I, II and IV after 1 hour of incubation with remifentanil at 50 and 500 ng/ml (n = 5 for 6a and b, and n = 4 for 6c) (C: control [respiration buffer alone], R50: remifentanil 50 ng/ml, R500: remifentanil 500 ng/ml). Data represent mean±SD. Statistical significance between samples using paired sample *t* test is indicated (*p<0.05 vs. control).

HepG2 cells' isolated mitochondria were incubated with remifentanil at 50 or 500 ng/ml (n = 5) for 1 hour (Figure 4). There were no significant changes in maximal mitochondrial respiration (state 3) for any of the complexes. However, remifentanil at a concentration of 500 ng/ml induced an increase in the respiratory control ratio (RCR) of complex-I-dependent respiration (1.89±0.6 in controls [medium alone] vs. 1.99±0.62 in stimulated cells; p = 0.028) (Figure 4c).

### Cellular oxygen consumption after incubating the cells with TNF-α and remifentanil

**Figure 5:**
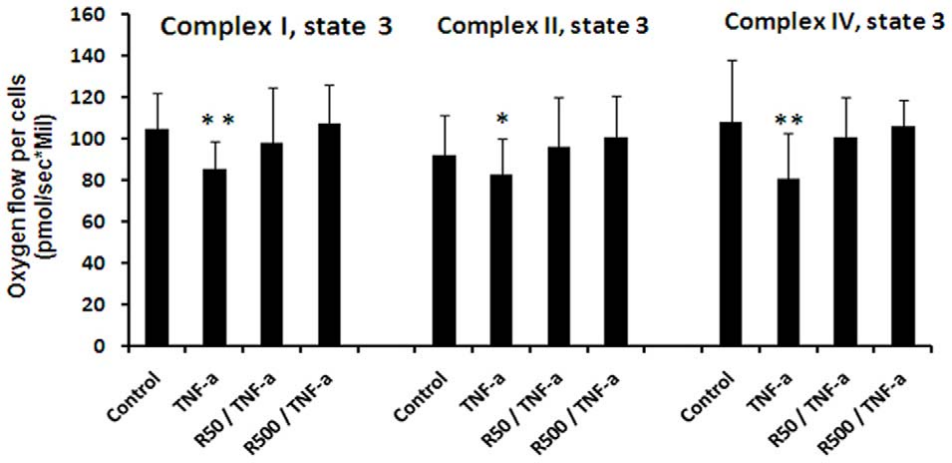
Cellular oxygen consumption after incubating the cells with TNF-α and remifentanil. HepG2 permeabilized cells' oxygen consumption for complex I, II and IV (state 3), incubated with medium alone (controls), TNF-α (10 ng/ml; 200 U/ml) for 1 hour, or preincubated with 50 ng/ml or 500 ng/ml remifentanil for 30 minutes followed by incubation with TNF-α (10 ng/ml, 200 U/ml) for an additional hour (n = 8). Each individual respiration experiment was performed in four chambers of high-resolution oxygraphs in parallel, one as a control and the others for the treated cells, and was recorded simultaneously for paired comparisons of the slopes of the oxygen concentrations of each individual experiment. (R50: remifentanil 50 ng/ml, R500: remifentanil 500 ng/ml). Data represent mean±SD. Statistical significance between samples using paired *t* test is indicated (*p<0.05 vs. control, **p<0.01 vs. control).

To determine whether remifentanil interferes with TNF-α-induced mitochondrial dysfunction, cells were treated for 30 min with medium alone or remifentanil (50 or 500 ng/ml), followed by incubation with TNF-α at 10 ng/ml (200 U/ml) for an additional hour (n = 8). Afterwards, cellular respiration was measured. Each individual respiration experiment was performed in four chambers of high-resolution oxygraphs in parallel, one as a control and the others for the treated cells, and was recorded simultaneously for paired comparisons of the slopes of the oxygen concentrations of each individual experiment. At 1 hour of TNF-α incubation, a significant reduction in respiration was observed for complex I-dependent (105±17 in controls vs. 86±13 pmoles/[s*million cells] in stimulated cells; p = 0.001), complex II-dependent (92±19 in controls vs. 83±17 pmoles/[s*million cells] in stimulated cells; p = 0.0037) and complex IV-dependent respiration (108±30 in controls vs. 81±22 pmoles/[s*million cells] in stimulated cells; p<0.001) (Figure 5). Preincubation of the cells with remifentnil at 50 ng or 500 ng for 30 min prevented TNF-α-induced decrease in mitochondrial respiration.

### Remifentanil attenuates TNF-α-induced activation of caspase-3 apoptosis signaling

**Figure 6:**
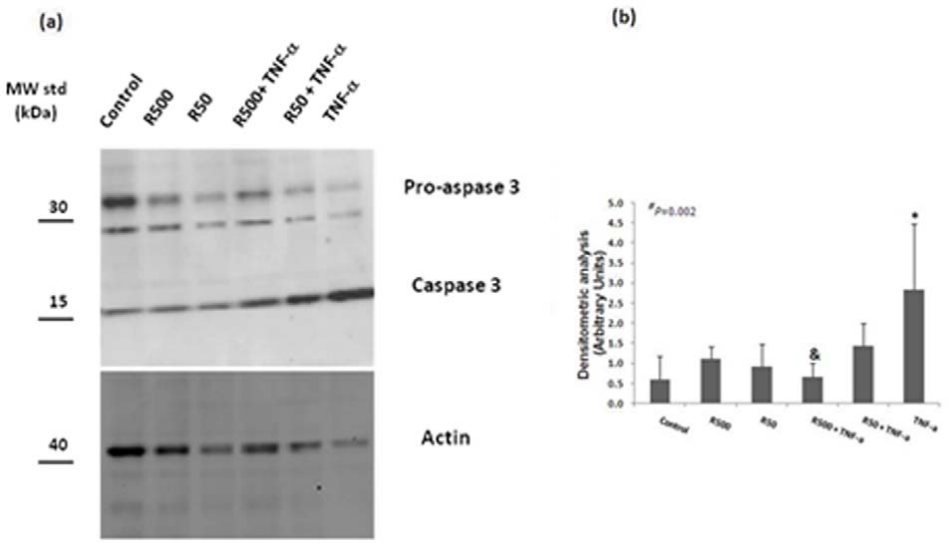
Remifentanil attenuates TNF-α-induced activation of caspase-3 apoptosis signalling. Western blot analysis of procaspase-3 and cleaved caspase 3 (panel a) from HepG2 cells after incubation with medium alone (controls), TNF-α (10 ng/ml) and remifentanil (50 and 500 ng/ml) for 24 h or preincubation with remifentanil (50 and 500 ng/ml, 1 h incubation) followed by incubation with TNF-α (10 ng/ml) for an additional 24 h (n = 5) (The polyclonal antibody used is specific to recognize both the precursor procaspase-3 ∼36 kDa and the mature active enzymes of 17–20 kDa following cleavage by upstream caspases) (C: control, R50: remifentanil 50 ng/ml, R500: remifentanil 500 ng/ml). Densitometric analysis of cleaved caspase protein levels (panel b): the protein bands were analyzed and quantified densitometrically using Quantity One software from Bio-Rad. The relative levels of cleaved caspase 3 expression were normalized to actin and the results are expressed as arbitrary units and are means+/−SD for five independent experiments. Statistical analysis for cleaved caspase 3 levels was performed by one-way analysis of variance (ANOVA): **^#^** p = 0.002. Afterwards, all groups were divided into subgroups and subsequent one-way ANOVA was performed in the subgroups (subgroup 1: control, TNF-α, R50 and R500 (p = 0.006); subgroup 2: TNF-α, R50+TNF-α and R500+TNF-α; p<0.016) followed by an independent t-test in each subgroup. * = independent t-test, TNF-α vs. control (p = 0.02). ^&^ = independent t-test R500+TNF- α vs. TNF-α (p = 0.02).

Cleavage of pro-caspase-3 into cleaved active caspase 3 serves as an early marker of apoptosis. To determine whether remifentanil attenuates TNF-α-induced activation of caspase-3 apoptosis signaling in HepG2 cells, we examined processing/activation of caspase-3 (Figure 6). For these experiments, cells were incubated with TNF-α at 10 ng/ml (200 U/ml) for 24 hours, or pretreated with 50 or 500 ng/ml of remifentil (for 1 hour), followed by incubation with medium alone or TNF-α at 10 ng/ml (200 U/ml) for an additional 24 hours. We used an antibody which can recognize both the inactive precursor procaspase-3 (∼36 kDa) and the mature active enzyme of ∼17 kDa following cleavage by upstream caspases. Treatment of the cells with TNF-α induced a reduction in procaspase-3 protein band intensity, with visible cleaved active caspase 3 fragments (densitometric analysis of cleaved caspase protein levels: 0.6±0.57 arbitrary units [AU] in controls vs. 2.84±1.62 AU in cells treated with TNF-α; p = 0.02) (n = 5). Pretreatment with remifentanil at a concentration of 500 ng/ml, followed by incubation with TNF-α, attenuated TNF-α-induced cleavage of caspase-3 (0.67±0.34 AU in cells pretreated with remifentanil [500 ng/ml] followed by incubation with TNF-α vs. 2.84±1.62 AU in cells treated with TNF-α; p = 0.02). However, pretreatment with remifentanil at a concentration of 50 ng/ml, followed by incubation with TNF-α, did not affect TNF-α-induced cleavage of caspase-3 (1.44±0.57 AU in cells pretreated with remifentanil [50 ng/ml] followed by incubation with TNF-α 2.84±1.62 AU in cells treated with TNF-α; p = 0.1).

### Cytokine release

**Figure 7:**
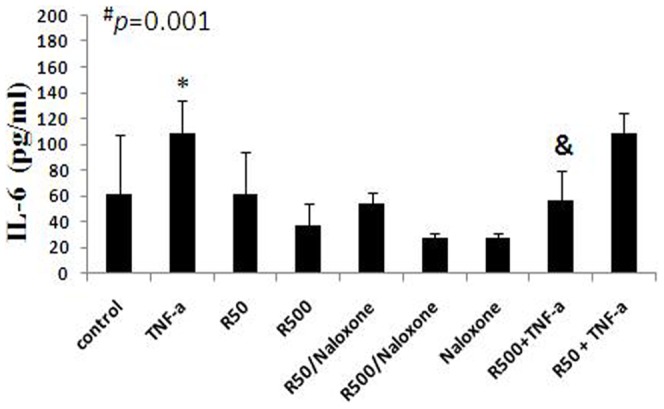
Modulation of the cytokine release. HepG2 supernatants' IL-6 protein content after 24 h of incubation with medium alone (controls), TNF-α (10 ng/ml) or remifentanil (50 and 500 ng/ml) for 24 h or preincubation with remifentanil (50 and 500 ng/ml, 1 h incubation) followed by incubation with TNF-α (10 ng/ml) for an additional 24 h. In addition, cells were pretreated with naloxone at 1000 ng/ml followed by incubation with 50 or 500 ng/ml of remifentil or medium alone for an additional 24 hour (n≥3) (C: control, R50: remifentanil 50 ng/ml, R500: remifentanil 500 ng/ml). Data represent mean±SD. Statistical analysis for cytokine levels was performed by one-way analysis of variance (ANOVA): **^#^** *p* = 0.001. Afterwards all groups were divided into subgroups and subsequent one-way ANOVA was performed in the subgroups (subgroup 1: control, TNF-α, R50, R500 and naloxone (p = 0.003); subgroup 2: R50, R500, R50+naloxone, R500+naloxone p = 0.081; subgroup 3: TNF-α, R50+TNF-α, R500+TNF-α, p = 0.006) followed by an independent t-test in each subgroup. * = independent t-test, TNF-α vs. control (p = 0.032). ^&^ = independent t-test R500+TNF-α vs. TNF-α (p = 0.008).

Supernatants of HepG2 cultures were measured for IL-6 and IL-10 contents after 24 hours of incubation with various agonists/antagonists (Figure 7). Treatment of HepG2 cells with TNF-α induced an increase in Il-6 levels (61.7±45.27 ng/ml in controls vs. 108.7±25.38 ng/ml in cells treated with TNF-α; p = 0.03). Pretreatment with remifentanil at a concentration of 500 ng/ml, followed by incubation with TNF-α, prevented TNF-α-induced IL-6 release (57±22.44 ng/ml in cells pretreated with remifentanil [500 ng/ml] followed by incubation with TNF-α; vs. 108.7±25.38 ng/ml in cells treated with TNF-α; p = 0.008). However, pretreatment with remifentanil at a concentration of 50 ng/ml, followed by incubation with TNF-α, did not affect TNF-α-induced IL-6 release (108.6±15.2 ng/ml in cells pretreated with remifentanil [50 ng/ml] followed by incubation with TNF-α; vs. 108.7±25.38 ng/ml in cells treated with TNF-α; p = 0.92).

IL-10 levels in the supernatants of HepG2 cells were undetectable in controls or cells treated with TNF-α, remifentanil, naloxone or combinations of remifentanil and naloxone/TNF-α (data not shown).

### Membrane potential, ATP content and coupled and uncoupled cellular respiration

**Figure 8:**
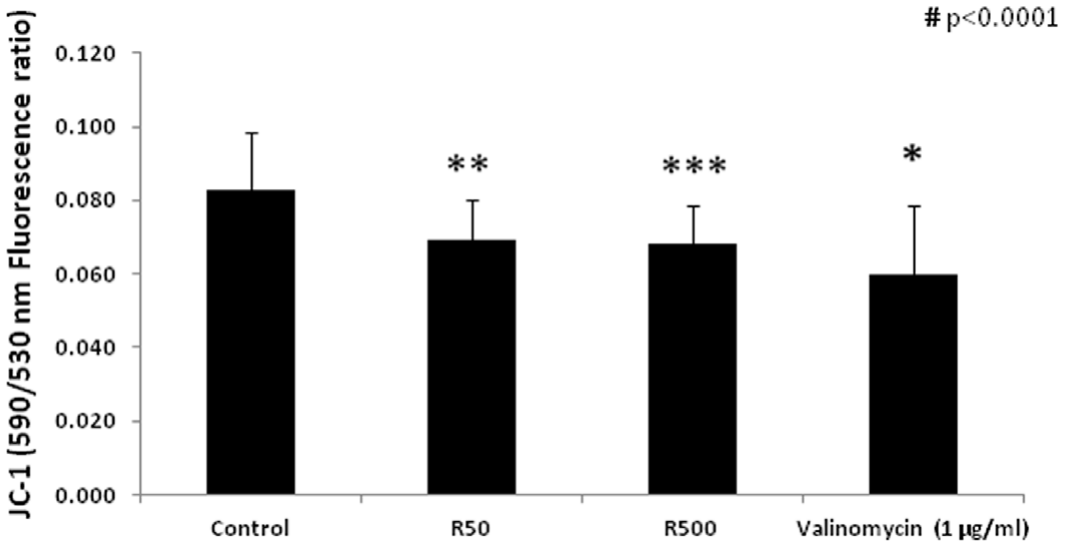
Mitochondrial membrane potential measured by the changes in the 590/530 JC-1 emitted fluorescence. HepG2 cells' mitochondrial membrane potential after incubation with medium alone for 1 hour (controls, n = 24), remifentanil at 50 (R50, n = 24) or 500 (R500, n = 32) ng/ml and valinomycin (1 µg/ml, n = 8)). Data represent mean±SD. Statistical analysis was performed using one-way analysis of variance (ANOVA): # p<0.0001, followed by an independent t-test (*p = 0.002 vs. control, **p = 0.001 vs. control and ***p<0.0001 vs. control).

Treatment of the cells with remifentanil at 50 and 500 ng/ml for 1 hour induced a reduction in mitochondrial membrane potential (Δψm depolarization), expressed as the reductions in JC-1 590/530 nm fluorescence ratios (0.083±0.016 in controls vs. 0.069±0.011 in cells treated with remifentanil at 50 ng/ml [p = 0.001] and 0.068±0.01 in cells treated with remifentanil at 500 ng/ml [p<0.0001]). Valinomycin (1 µg/ml, 1 hour incubation), which was used as positive control, also dissipated the mitochondrial electrochemical potential (0.060±0.019 vs controls, p = 0.002) (Figure 8).

**Figure 9:**
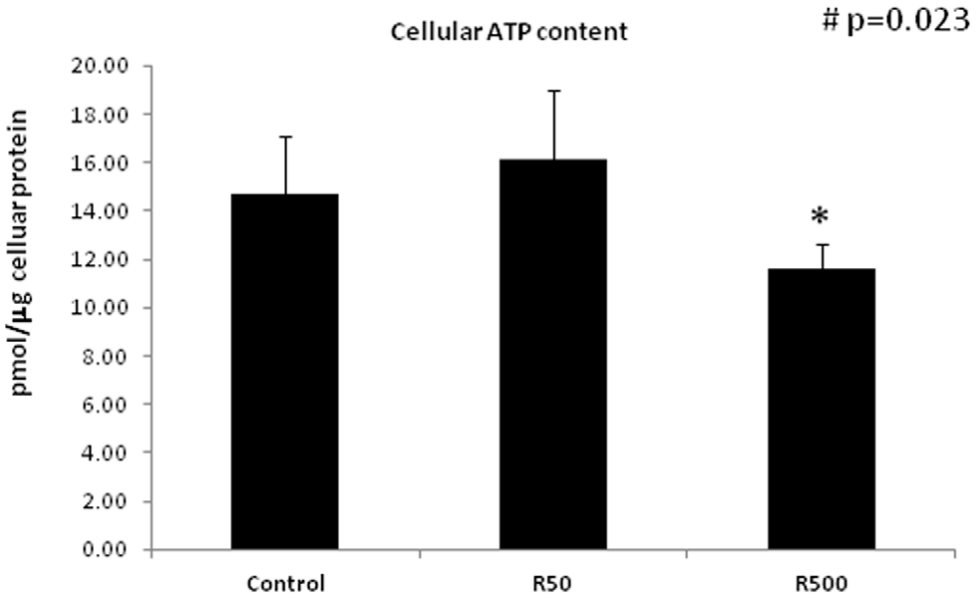
HepG2 cells' mitochondrial ATP. HepG2 cells' mitochondrial ATP content after incubation with medium alone (controls) or with remifentanil at 50 (R50) or 500 (R500) ng/mlor for 1 hour. Data represent mean±SD. Statistical analysis was performed using one-way analysis of variance (ANOVA): # p = 0.023, followed by an independent t-test (*p = 0.031 vs. control) (n = 5).

To assess whether reduction in the mitochondrial membrane potential (50 and 500 ng/ml) was accompanied by a reduction in cellular ATP levels, we measured the ATP content of the cells after treatment with remifentanil (50 or 500 ng/ml). Treatment with remifentanil for 1 hour led to a reduction in cellular ATP content only at 500 ng/ml (11.63±1.04 pmole/µg cellular protein in remifentanil 500 ng/ml treated cells vs. 14.69±2.39 pmole/µg in controls, p = 0.023) (Figure 9).

**Figure 10:**
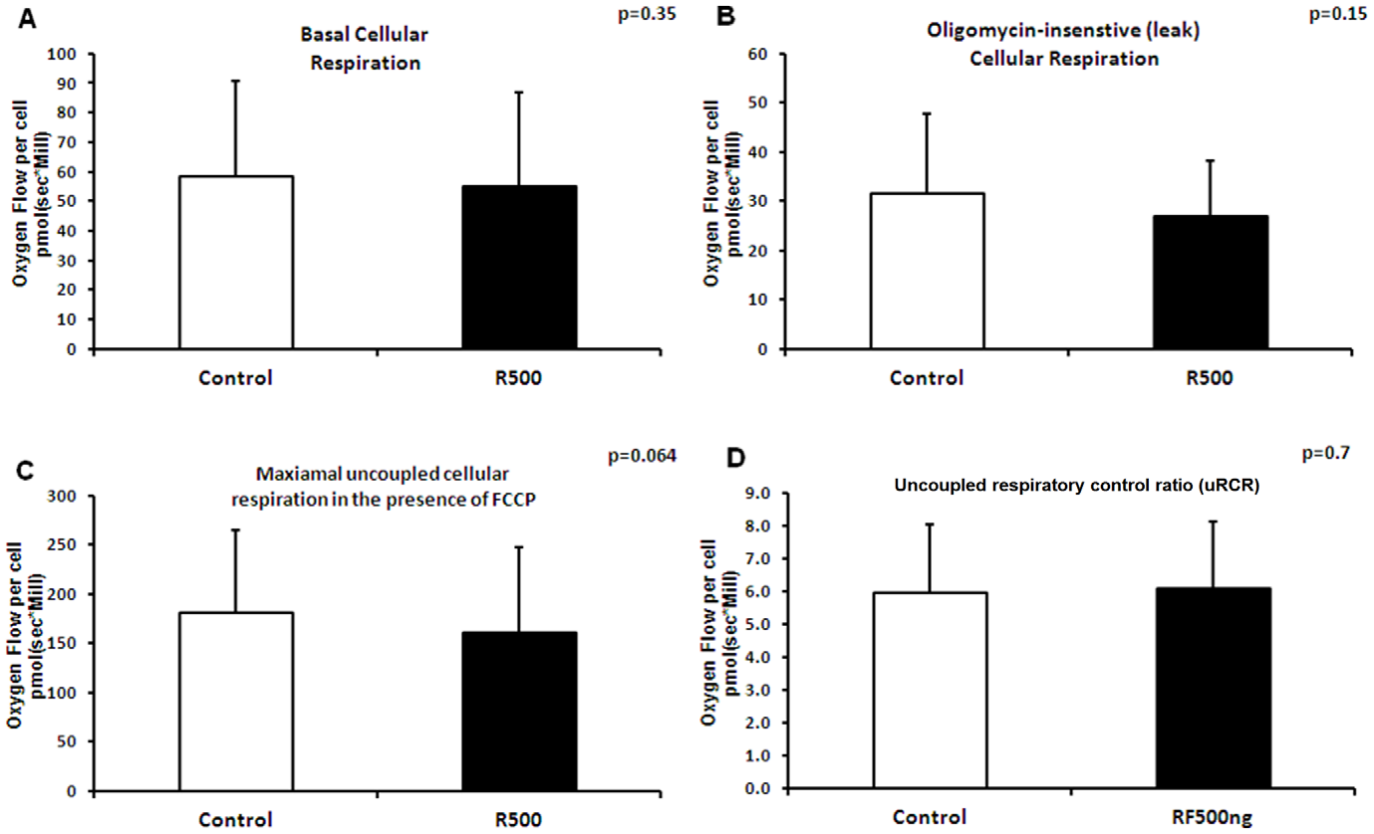
Intact cellular oxygen consumption after incubation with remifentanil. Intact cellular oxygen consumption after incubation with or without remifentanil at 500 ng/ml for 1 hour. HepG2 cells' basal oxygen consumption (A), in the presence of oligomycin (B: oligomycin insensitive respiration), and FCCP (C) (n = 20 each). (C: control [medium alone], R: remifentanil)(n = 20). The uncoupled respiratory control ratio (uRCR) was calculated as the ratio between the oxygen consumption rate in the presence of FCCP and the rate in the presence of oligomycin (D). Data represent mean±SD. Statistical significance between samples using paired samples *t* test is indicated.

Remifentanil at 500 ng/ml increased complex I- and II-dependent respiration of permeabilized cellular respiration (using exogenous substrates), and decreased cellular ATP content and membrane potential, indicating that at high dosage it might act as a mitochondrial uncoupler. Therefore we performed additional experiments investigating the overall endogenous respiration of intact cells in the absence of exogenous substrates and ADP. For these experiments, cell were treated with remifentanil (500 ng/ml, 1 hour incubation) and endogenous basal cellular respiration, FCCP uncoupled and oligomycin-insensitive respiration rates were measured. Treatment of the cells with remifentanil (500 ng/ml, 1 hour incubation) did not affect basal endogenous cellular respiration of intact cells (58.5±32.4 in controls vs. 54.9±32.2 in cells treated with remifentanil [p = 0.35] Figure 10A). FCCP-uncoupled maximal respiration rates (respiration in the presence of FCCP) tended to decrease (180.4±85.2 in controls vs. 161.0±86.1 cells treated with remifentanil p = 0.064) (Figure 10C), indicating a tendency in decreased respiratory capacity after treatment with remifentanil. Oligomycin-insensitive respiration which represents non-phosphorylating respiration (Figure 10B), Oligomycin-sensitive respiration (ATP turnover; data not shown) and uncoupled RCRs (uRCRs: the ratio between FCCP and oligomycin-insensitive respiration rates; 6.0±2.1 in controls vs. 6.1±2.0 in cells treated with remifentanil [p = 0.7]) were not affected (Figure 10D).

## Discussion

In the current study, we demonstrate that in cultured human hepatocytes, remifentanil improves mitochondrial bioenergetics and prevents TNF-α-induced reduction of mitochondrial respiration.

We selected three different concentrations of remifentanil. At a clinical and therapeutic blood concentration of 50 ng/ml [22], [32], remifentanil increased complex-I-dependent mitochondrial respiration. At 10 times higher than therapeutic concentration (500 ng/ml), remifentanil increased complex I- and II-dependent respiration, indicating that higher doses of this drug lead to greater alterations in mitochondrial bioenergetics. At one tenth of therapeutic concentration (5 ng/ml), remifentanil did not affect mitochondrial respiration.

Early on (within the first hour of incubation) we observed remifentanil-induced increases in maximal cellular respiration, an effect which was not present at later time points. This can be explained by the ultra-short pharmacokinetic profile of the drug [22]–[24] and the fact that in the present study remifentanil was not administered continuously to the cell culture flasks. Remifentanil exhibits rapid metabolism by non-specific esterases in the tissues, principally to a carboxylic acid derivative, remifentanil acid (RA). In patients treated with remifentanil, this carboxylic acid derivative is eliminated by the kidneys; however, in our experiments it should have accumulated in cell culture flasks. Therefore we extended the mitochondrial respiration experiments to up to 16 hours to ensure that the accumulated carboxylic acid derivative of remifentanil does not interfere with cellular respiration. One limitation of our study was that we did not measure concentration-time profiles of remifentanil and its metabolized carboxylic acid derivative in the cell culture flasks.

Remifentanil incubation at a concentration of 500 ng/ml induced a significant increase in maximal exogenous ADP-stimulated respiration (state 3) of permeabilized cells, using excess substrates suggesting altered mitochondrial bioenergetics. State 3 respirations represent oxidative phosphorylation capacity of the pemeabilized cells (or isolated mitochondria) in the presence of saturating ADP and excess substrates. Maximal oxygen consumption in state 3 is a combination of coupled and uncoupled respiration (due to physiological or pathological conditions). To investigate whether the observed increase in maximal respiration of HepG2 cells treated with remifentanil is the result of mitochondrial respiration uncoupling, we performed additional respiratory experiments in intact cells. For these experiments, we measured intact cellular basal endogenous respiration. To evaluate the respiratory electron transfer system capacity, non-coupled respiration was induced experimentally by titration of FCCP to obtain the maximum flux. We did not observe any difference in basal (endogenous) respiration of cells treated with remifentanil at 500 ng/ml compared to controls. Basal respiration of the intact cells in the absence of exogenous substrates or ADP mainly reflects cellular ATP requirement to maintain a basal metabolic rate and not respiratory capacity [33]. Basal respiration of intact cells does not represent saturating ADP-stimulated state 3 respiration and is substrate limited. We further measured FCCP-uncoupled respiration, which is a measure of the mitochondrial electron transport chain capacity and integrity. Remifentanil induced a tendency to reduce the maximum electron transport chain capacity of intact cells. However, the uncoupled respiratory control ratio (uRCR) which is the ratio of the FCCP uncoupled maximal respiration to oligomycin-insensitive respiration, reflecting respiratory reserve capacity, was not affected. We did not observe any difference in state 4 respiration of isolated HepG2 mitochondria treated with remifentanil at 500 ng/ml compared to controls indicating absence of uncoupling. The present data suggest that remifentanil does not uncouple HepG2 cells mitochondrial respiration. A theoretical alternative explanation for the measured effects of remifentanil is activation of mitochondrial ATP-sensitive K+ (mtKATP) channel. Activation of mtKATP channel can play an essential role in activation the respiratory chain [34]–[38]. It has been proposed that opening the mtKATP channel can lead to increased mitochondrial matrix volume, increased respiration and induces a slight reduction in mitochondrial membrane potential [39]. In isolated rat heart mitochondria, mtKATP channel opening depolarized mitochondrial membrane potential, increased respiration, slowed ATP production, and increased matrix volume [40].

Since remifentanil is an opioid acting on μ-type receptors [22] and expression of the μ-opioid receptor in hepatocytes has been reported previously [41], we investigated whether the μ-opioid activity of remifentanil could be antagonised by a narcotic antagonist, naloxone. Preincubation with naloxone attenuated remifentanil-induced increases in mitochondrial respiration, indicating that the remifentanil effect was mediated by an opioid receptor mechanism. Furthermore, we observed remifentanil-induced phosphorylation of IκBα early (within the first 30 min), denoting the stimulation of NF-κB. Activation of the NF-kB transcription factor by the μ-opioid-receptor agonist and signaling has been demonstrated [42], and our data again indicate activation of opioid receptor signaling.

Since it has been shown that anaesthetic drugs might have a direct effect on isolated mitochondrial oxygen consumption and bioenergetics [43], we isolated mitochondria from HepG2 cells and treated them with remifentanil. At the dosage (50 or 500 ng/ml) used, remifentanil did not interfere with isolated mitochondrial maximal respiration (state 3) of HepG2 cells, but at 500 ng/ml it induced an increase in the respiratory control ratio of complex I-dependent respiration, indicating an increase in respiratory activity of the isolated mitochondria. Our data are in disagreement with a study in which the authors observed a reduction in isolated mitochondrial respiration for mitochondria preincubated with remifentanil [44]. However, in their study the authors used an extremely high dose of remifentanil (10,000 to 20,000 ng/ml) in a different organ (brain) and species (rat).

As mentioned above, remifentanil is an opioid acting on cell surface μ-type receptors [22], and the mechanism through which remifentanil has a direct effect on isolated mitochondrial respiration should be investigated in more detail. Remifentanil might be able to cross the cellular membrane and reach mitochondria. One can speculate that remifentanil might have a direct effect on mitochondrial enzymes located in the inner mitochondrial membranes by interacting with respiratory chain complexes or may affect proton leakage by binding to the mitochondrial membrane. While we cannot exclude impaired function of mitochondria due to the isolation procedure**,** maintained quantitative oxygen kinetics using high-resolution respirometry has been demonstrated [45], and measurements in isolated mitochondria remain one of the gold standards in studies addressing mitochondrial physiology.

TNF-α is one of the important mediators of the inflammatory response seen in sepsis/septic shock and may also induce mitocondrial dysfunction in human hepatocytes [31]. We investigated further whether remifentanil interferes with the effects of TNF-α on mitochondrial respiration. We observed that remifentanil prevented TNF-α-induced mitochondrial dysfunction of cultured hepatocytes. The regulatory immune functions of μ-opioid receptor activators have also been shown in several animal models of inflammatory diseases [46], [47]. In one study, the opioid agonist morphine inhibited LPS-induced TNF-*α* production in vivo [48]. Chakass et al. showed that μ-opioid receptor activation prevented acute hepatic inflammation and cell death, and the administration of a selective μ-opioid receptor agonist enhanced hepatoprotective-signaling pathways in vivo [41]. Recently we showed that pretreatment of cultured hepatocytes with cyclosporine A prevented TNF-α-induced reduction of cellular respiration, implicating the involvement of the mitochondrial permeability transition pore openings [31]. Interestingly, in a recent study the opioid agonist morphine prevented the mitochondrial permeability transition pore opening in cardiomyocytes [49]. One can speculate that remifentanil has the same mode of action as morphine, by acting on the mitochondrial permeability transition pore openings.

We further observed anti-apoptotic effects of remifentanil by attenuating TNF-α-induced fragmentation of pro-caspase-3 into cleaved active caspase 3. Chakass et al. also showed that treatment with μ-opioid receptor agonist decreased ceramide-induced cell death in cultured human hepatocytes, indicating an anti-apoptotic effect of μ-opioid receptor agonists [41].

IL-6 plays an important role in innate and acquired immune responses [50] and is up-regulated during sepsis [51], [52]. In humans, serum levels of IL-6 are increased after TNF-α infusion [53], [54]. Opioids can modulate immune responses, and it has been reported previously that remifentanil could prevent endotoxin-induced cytokine release in human whole blood cells in vitro [55]. We therefore exposed the cells under septic conditions, by incubating with TNF-α to induce IL-6 expression and release in cellular supernatants, and investigated whether remifentanil could prevent TNF-α-induced cytokine release. We showed that remifentanil at a high dose prevented TNF-α-induced Il-6 release.

A limitation of our study is the use of HepG2 cells, which show certain dissimilarities when compared to cells obtained directly from patients [56]. For instance, despite similar glucuronide conjugation compared to human hepatocytes, lower dealkylation activity and higher microsomal hydrolysis have been reported [57]. How this may have affected metabolism of the various compounds used in our study is difficult to judge. Accordingly, the relevance of our findings for patients should be further investigated.

In summary, our data show that remifentanil induced an increase in maximal oxygen consumption of cultured human hepatocytes which was prevented by preincubation with an opioid receptor antagonist, indicating that remifentanil's effect was mediated by an opioid receptor mechanism**.** Remifentanil did not interfere with isolated mitochondrial maximal respiration (state 3) of HepG2 cells, but it increased the respiratory control ratio of complex-I-dependent respiration. Preincubation of the cells with remifentanil prevented TNF-α-induced mitochondrial dysfunction and attenuated TNF-α-induced fragmentation of pro-caspase-3 into cleaved active caspase 3 and IL-6 release of HepG2 cells.

The magnitude of the effect of remifentanil on mitochondrial respiration was small, and is most likely not clinically relevant under normal conditions. Nevertheless, effects on mitochondrial respiration of a similar magnitude have been reported for other commonly used drugs such as nitroglycerin [58], metformin [59] and various catecholamines [60]. Accordingly, drug-induced mitochondrial alterations can be potentiated by jointly used drugs with similar effects. Moreover, patients with mitochondrial disorders may have a more exaggerated response when exposed to drugs which normally interfere minimally with mitochondrial functions.

## Materials and Methods

### Chemicals and reagents

Remifentanil was obtained from Glaxo-Wellcome AG (Bern, Switzerland) and naloxone from OrPha Swiss GmbH (Küsnacht, Switzerland). All the reagents for cellular respiration and media for cell culture, as well as recombinant human TNF-α, polyclonal anti-actin antibody, and cyclosporin A (CsA), were obtained from Sigma-Aldrich (Buchs, Switzerland). Caspase-3 antibody was purchased from Assay Designs (Ann Arbor, MI, USA) and horseradish peroxidase anti-rabbit secondary antibody from Abcam (Cambridge, UK).

### Cell culture

The human hepatoma cell line HepG2 was cultured in 25 cm^2^ flasks (for respiration assays) or 24-well plates (for Western blot analysis) in RPMI 1640 containing 10% heat-inactivated fetal bovine serum (FBS), 1% non-essential amino acids, 1% glutamine, 1% sodium pyruvate, and 1% penicillin-streptomycin at 37°C in a humid atmosphere (5% CO_2_, 95% air), with passage twice a week. Quiescent cells were obtained by total deprivation of FBS for 14 to 16 hours before the experiments. All experiments were performed when cells reached 90–95% confluency. Cells were exposed to remifentanil at 5 or 500 ng/ml for 1 hour, at 50 ng/ml for 1, 2, 4, 8 and 16 hours, or pretreated with naloxone at 1000 ng/ml for 1 hour, followed by incubation with remifentanil at 50 or 500 ng/ml for an additional hour. In an additional series of experiments, cells were incubated with TNF-α at 10 ng/ml (200 U/ml) for 1 hour or pretreated with 50 or 500 ng/ml of remifentil, followed by incubation with TNF-α at 10 ng/ml (200 U/ml) for an additional hour.

### Mitochondrial isolation

Mitochondria were isolated from cultured human hepatocytes by a method of homogenisation with a Dounce homogeniser (20–30 strokes), followed by low-speed (600 *g*) and high-speed (11,000 *g*) centrifugations using a Mitochondria Isolation Kit (Sigma, Switzerland) according to the manufacturer's instructions.

### Cellular respiration (high-resolution respirometry)

After incubation, HepG2 cells were trypsinised and resuspended in RPMI-1640 with 10% FBS, and then centrifuged for 5 min (350 g). Cells were resuspended in the respiration buffer [61] (110 mM sucrose, 0.5 mM EGTA, 3.0 mM MgCl2, 80 mM KCl, 60 mM K-lactobionate, 10 mM KH2PO4, 20 mM taurine, 20 mM hepes, 1.0 g/l BSA, pH 7.1) at a concentration of 1- 2×10^6^ cells/ml. Respiration rates were measured at 37°C using a high-resolution oxygraph (Oxygraph-2k, Oroboros Instruments, Innsbruck, Austria). Respiration rates were calculated as the time derivative of oxygen concentration measured in the closed respirometer and expressed per million viable cells. The amplified signal was recorded in a computer with online display of the calibrated oxygen concentration and oxygen flux (DatLab software for data acquisition and analysis; Oroboros Instruments, Innsbruck, Austria). Mitochondrial complex activity was assessed by a standard titration protocol: first cells were permeabilized with digitonin (8.1 µM) for 5 min. Afterwards, for complex I-dependent maximal respiration stimulation, substrates added were glutamate (10 mM) and malate (5 mM), which provide nicotinamide adenine dinucleotide (NADH) to the respiratory chain (complex I activation), followed by addition of ADP (2.5 mM) (state 3, maximal respiration). After a stable signal was reached and marked, rotenone (0.5 µM) was added to inhibit complex I, and then complex II-dependent respiration was stimulated by adding succinate (10 mM), which provides flavin adenine dinucleotide (FADH) to the respiratory chain (complex II activation, state 3). Afterwards, complex III was inhibited by antimycin A (0.5 µM), and complex IV-dependent respiration was measured by adding ascorbate (4 mM) and N,N,N′,N′-tetramethyl-p-phenylendiamine (TMPD, 0.5 mM). Since TMPD exhibited a wide range of auto-oxidation in the buffer, respiration was finally inhibited with sodium azide (5 mM), and the difference between the oxygen consumption before and after the addition of sodium azide was interpreted as the real complex IV respiration.

Each individual respiration experiment was performed in two to four chambers in parallel, one as a control and the others for the treated cells, and was recorded simultaneously for paired comparisons of the slopes of the oxygen concentrations of each individual experiment.

### Isolated mitochondrial oxygen consumption

Isolated HepG2 cells' mitochondria (isolated from 20 to 40 75 cm^2^ cultured flasks) were incubated with remifentanil at 50 or 500 ng/ml for 1 hour. Isolated mitochondria were resuspended in the respiration buffer (same buffer used for permeabilized cells) at a concentration of 0.4 mg/ml, and respiration rates were measured at 37°C with the high-resolution respirometer. The medium was equilibrated for 30 to 40 minutes with air in the oxygraph chambers and stirred at 750 rpm until a stable signal was obtained for calibration at air saturation. The corresponding oxygen concentration was calculated from the digitally recorded barometric pressure and the oxygen solubility at 37°C. The amplified signal was recorded in a computer with online display of the calibrated oxygen concentration and oxygen flux (negative time derivative of oxygen concentration; Dat-Lab software for data acquisition and analysis; OROBOROS). Oxygen consumption was expressed as pmol/second/mg mitochondrial protein. Oxygen levels were always maintained above 40 nmol/ml. Maximal oxidative capacities were determined in the presence of saturating concentrations of oxygen, ADP (0.25 mmol/l) and specific mitochondrial substrates. For complex I-dependent respiration, substrates were glutamate (10 mmol/l) plus malate (5 mmol/l), which provide nicotinamide adenine dinucleotide (NADH) to the respiratory chain (complex I activation). For measurement of complex II-dependent respiration, first complex I was inhibited with rotenone (0.5 µmol/l), and then succinate (10 mmol/l) was added, which provides flavin adenine dinucleotide to the respiratory chain (complex II activation). The coupling of phosphorylation to oxidation was determined by calculating the respiratory control ratio (RCR) as the ratio between ADP-stimulated respiration (state 3) and respiration after ADP depletion (state 4). Complex IV-dependent respiration was measured by adding ascorbate (4 mM) and N,N,N′,N′-tetramethyl-p-phenylendiamine (TMPD, 0.5 mM). Since TMPD exhibited a wide range of auto-oxidation in the buffer, respiration was finally inhibited with sodium azide (5 mM), and the difference between the oxygen consumption before and after the addition of sodium azide was interpreted as the real complex IV (state 3) respiration.

### Measurement of phosphorylation of IκBα

To demonstrate that the remifentanil effect was mediated by a cell surface opioid receptor, we measured phosphorylation and activation of IκBα. Detection and analysis of IκBα phosphorylation of cell extract of remifentanil-induced (50 and 500 ng/ml, for 30 min) HepG2 were performed with a FunctionELISA^TM^ IκBα assay kit (Active Motif, Carlsbad, CA, USA) according to the manufacturer's instructions.

### Sodium dodecyl sulphate polyacrylamide gel electrophoresis (SDS-PAGE) and Western blotting

SDS-PAGE and immunoblot analysis were performed as described previously [31]. Briefly, for immunoblot analysis of caspase 3, quiescent cells (cultured in 24-well plates) obtained by total deprivation of FBS for 14 to 16 hours before the experiments, were incubated with TNF-α at 10 ng/ml (200 U/ml) for 24 hours or pretreated with 50 or 500 ng/ml of remifentil for 1 hour, followed by incubation with medium alone or TNF-α at 10 ng/ml (200 U/ml) for an additional 24 hours. Afterwards, the cells were lysed in 60 mM Tris-HCl, 8.5% glycerol, and 2% SDS. The protein concentration was determined with the Quant-iTM assay kit and read with the Qubit-TM fluorometer (Invitrogen®, Basel, Switzerland). Equal amounts of protein (20 µg per line) were loaded and separated by 4–12% SDS-PAGE. Gels were then transferred into nitrocellulose membranes with the iBlot-TM dry blotting system (Invitrogen®, Basel, Switzerland). Equal loading was verified by staining the exgel with Simply Blue-TM SafeStain (Invitrogen®, Basel, Switzerland). Afterwards, the membranes were blocked for 30 min with the incubation buffer (10 mM Tris-HCl pH 7.5, 100 mM NaCl and 0.1% W/V Tween 20) supplemented with 5% non-fat dry skim milk, and then incubated overnight with the primary antibodies: polyclonal caspase-3 antibody (dilution 1∶1000; the antibody recognizes both the precursor procaspase-3 ∼36 kDa and the mature active enzymes of 17–20 kDa following cleavage by upstream caspases) and anti-actin antibody (dilution 1∶3000). The membranes were then washed three times with incubation buffer and incubated for 1 hour with horseradish peroxidase goat polyclonal anti-rabbit IgG (dilution 1∶3000). Afterwards, membranes were developed with the enhanced chemiluminescence detection kit (Pierce, Rockford, IL, USA). Cleaved caspase-3 protein levels (bands) were analyzed and quantified densimetrically by using Quantity One software from Bio-Rad. The relative levels of cleaved caspase 3 expression were normalized to actin, and the results are expressed as arbitrary units (means+/−SD). All Western blotting experiments were performed in quintuplicate.

### Enzyme-linked immunosorbent assay (ELISA)

To investigate whether incubation with TNF-α induced the expected pro-inflammatory (IL-6) and anti-inflamatory (IL-10) cytokine release, and whether this effect could also be prevented by remifentanil, IL-6 and IL-10 contents in cell supernatants were measured using the LEGEND MAX™ Human ELISA Kit (Biolegend, San Diego, CA, USA) according to the manufacturer's instructions. For ELISA, cells were incubated with TNF-α at 10 ng/ml (200 U/ml) for 24 hours or pretreated with 50 or 500 ng/ml of remifentil, followed by incubation with medium alone or TNF-α at 10 ng/ml (200 U/ml) for an additional 24 hours. In addition, cells were also pretreated with naloxone at 1000 ng/ml, followed by incubation with 50 or 500 ng/ml of remifentil or medium alone for an additional 24 hours.

### Measurement of mitochondrial membrane potential in intact cells

The mitochondrial electrochemical potential gradient (Δψm) in intact cells was measured using the cationic dye JC-1 (5,5′,6′,6′-tetrachloro-1,1′,3,3′-tetraethylbenzimidazolocarbocyanine iodide). JC-1 is a mitochondrial sensor which aggregates in polarized mitochondria, where it forms red fluorescent aggregates (J-aggregates). Dissipation of the mitochondrial membrane potential prevents the accumulation of the JC-1 dye in the mitochondria, and the dye is dispersed throughout the entire cell, leading to a shift from red (J-aggregates) to green fluorescence (JC-1 monomers). Thus the loss of JC-1 aggregates directly correlates with changes in Δψm. Briefly, for these experiments, the cells were grown in 96-well plates and treated with remifentanil for 1 hour (50 and 500 ng/ml), and the mitochondrial membrane potential was measured using the JC-1 mitochondria staining kit for mitochondrial potential change detection (Sigma, Switzerland) according to the manufacturer's instructions. Cells serving as positive controls were treated with valinomycin (1 µg/ml, for 1 hour), which dissipates the mitochondrial electrochemical potential. Δψm was measured immediately by fluormetry. For JC-1 monomers, the fluorimeter was set at a 490 nm excitation wavelength and 530 nm emission wavelength and fluorescence was measured. For JC-1 aggregates, the fluorimeter was set at a 525 nm excitation wavelength and 590 nm emission wavelength and fluorescence was measured. Afterwards, the Δψm (590/530 nm fluorescence ratio) was calculated.

### Measurement of the HepG2 cells ATP content

Detection and analysis of total cellular ATP content of remifentanil-treated (50 and 500 ng/ml, for 1 hour) HepG2 cells were performed using the ATP determination kit (Invitrogen®, Basel, Switzerland) according to the manufacturer's instructions.

### Coupled and uncoupled respiration of intact cells

After incubation with remifentanil (500 ng/ml, 1 hour), HepG2 cells were trypsinised and resuspended in RPMI-1640 with 10% FBS, and then centrifuged for 5 min (350 g). Cells were resuspended in the respiration buffer (110 mM sucrose, 0.5 mM EGTA, 3.0 mM MgCl2, 80 mM KCl, 60 mM K-lactobionate, 10 mM KH2PO4, 20 mM taurine, 20 mM hepes, 1.0 g/l BSA, pH 7.1) at a concentration of 1- 2×10^6^ cells/ml. Respiration rates were measured at 37°C using a high-resolution oxygraph (Oxygraph-2k, Oroboros Instruments, Innsbruck, Austria). Basal coupled endogenous respiration of intact cells (oxygen consumption without the addition of exogenous substrate) was measured and recorded using the linear rate of oxygen consumption. Afterwards, oligomycin (an inhibitor of ATP synthase) (0.4 µg/ml) was added and the non-phosphorylating respiration rate was measured (oligomycin-insensitive respiration). Afterwards the chemical uncoupler FCCP *(carbonyl cyanide p-trifluoromethoxyphenylhydrazone)* was sequentially added at different concentrations (0.1 to 0.8 µM) and maximal uncoupled respiration was recorded. The uncoupled respiratory control ratio (uRCR) was calculated as the ratio between the oxygen consumption rate in the presence of FCCP and the rate in the presence of oligomycin. The oligomycin-sensitive respiration (ATP turnover) was calculated by subtracting the oligomycin-insensitive respiration from basal endogenous respiration.

### Statistics

The SPSS 15.0 software package (SPSS Inc®, Chicago, IL, USA) was used for statistical analysis. Comparisons of the slopes of the oxygen concentrations (cellular respiration) of each individual experiment were made using the paired samples *t* test. Unpaired Student's *t* tests were performed for evaluation of significance of phosphorylation of IκBα. Statistical analysis of cytokine levels (log-transformed values to normalize the data distribution) and densitometric analysis of cleaved caspase protein levels were performed by one-way analysis of variance (ANOVA). Afterwards the groups were divided into subgroups and subsequent one-way ANOVA was performed in the subgroups (for cytokines: subgroup 1: control, TNF-α, remifentanil 50 ng/ml [R50], remifentanil 500 ng/ml [R500], and naloxone; subgroup 2: remifentanil 50 ng/ml [R50], remifentanil 500 ng/ml [R500], remifentanil 50 ng/ml [R50]+naloxone, remifentanil 500 ng/ml [R500]+naloxone; subgroup 3: TNF-α, remifentanil 50 ng/ml [R50]+TNF-α, remifentanil 500 ng/ml [R500]+TNF-α, and for densitometric analysis of cleaved caspase: subgroup 1: control, TNF-α, remifentanil 50 ng/ml [R50] and remifentanil 500 ng/ml [R500], and subgroup 2: TNF-α, remifentanil 50 ng/ml [R50]+TNF-α and remifentanil 500 ng/ml [R500]+TNF-α) followed by an independent t-test. All data are presented as mean±SD, and a p<0.05 was considered significant.
