# Correction: Effect of Remifentanil on Mitochondrial Oxygen Consumption of Cultured Human Hepatocytes

**DOI:** 10.1371/annotation/1165f27b-aec3-4bbc-b846-56a3dbe31f7b

**Published:** 2012-09-27

**Authors:** Siamak Djafarzadeh, Madhusudanarao Vuda, Jukka Takala, Stephan M. Jakob

There was an error in the published Funding statement. The correct Funding is: 

This study was supported by the Swiss National Science Foundation (Grant nº 32003B_127619) and by funds from the Department of Intensive Care Medicine, University Hospital and University of Bern. The funders had no role in study design, data collection and analysis, decision to publish, or preparation of the manuscript. 

